# US perspective

**DOI:** 10.1186/1470-7330-15-S1-O3

**Published:** 2015-10-02

**Authors:** Hebert Alberto Vargas

**Affiliations:** 1 Department of Radiology, Memorial Sloan Kettering Cancer Center, 1275 York Avenue, New York, NY 10065, USA

## 

There are many key elements necessary to maximize the clinical utility of diagnostic imaging exams, including a pertinent clinical indication, adequate technical acquisition, accurate interpretation and effective communication of the imaging findings. The literature suggests that structured reporting in radiology leads to clearer and more thorough communication of relevant diagnostic findings than does conventional, free-form reporting. In a study of body oncologic CT examinations, structured reports were given significantly higher satisfaction ratings by both radiologists and referring physicians compared to “free-form” reports [1]. Barbosa et al. found that in addition to being preferred by the majority of the radiologists and endocrinologists participating in a study evaluating thyroid ultrasounds, the use of structured reporting resulted in improved standardization of thyroid finding descriptors [2]. A study of coronary CT angiograms found an improved inter-observer agreement for the number of vessels with significant stenosis when a structured reporting software which required the radiologist to explicitly state which vessels were involved was used [3]. Other structured reporting software with features such as drop-down menus which facilitate data entry and minimize the amount of free-text entries have been shown to aid not only data comprehension but also reduce the length of time required for aortic aneurysm imaging [4].

However, the benefits of structured reporting cannot be accepted dogmatically. An accurate interpretation reported in “free-form” style is more clinically useful than a structured report containing erroneous information. Furthermore, the terminology used in structured reports also requires standardization. Khorasani et al reported poor agreement between radiologists and non-radiologists in the interpretation of the most commonly used phrases in radiology reports [5]. In recent surveys gathering opinions about radiology reports, 20% of the responding clinicians indicated that they found the language and style of radiology reports unclear [6]. Another study found that referring clinicians may reach different conclusions when reading the same reports [7].

Another important issue relevant to standardized reporting is the expression of diagnostic certainty. Radiologists are often tasked with summarizing multiple findings and rendering an opinion with regards to potential explanations for the radiographic findings. There are scenarios in which no differential diagnoses are warranted and the findings are reported in terms of the absolute presence or absence of a pathologic process (e.g. “no fracture”). In other cases the findings are not definitive, and radiologists need to indicate their level of certainty for their interpretation of the imaging findings. In a study of patients with prostate cancer, 38 different terms were used in MRI reports to express the levels of certainty for the presence of extracapsular extension, prior to the introduction of a 5-point “certainty lexicon” [8]. The lexicon not only simplified the communication of the radiologists' level of suspicion but also allowed more objective quantification of the diagnostic performance of MRI for diagnosing ECE, with a reported area under the curve of 0.85 [8]. The development of standardized “lexicons” to indicate the radiologists' level of certainty for interpreting the imaging findings should therefore be considered an integral component of structured reports.
